# Nanosensors-Assisted Quantitative Analysis of Biochemical Processes in Droplets

**DOI:** 10.3390/mi11020138

**Published:** 2020-01-26

**Authors:** Dmitry Belyaev, Julian Schütt, Bergoi Ibarlucea, Taiuk Rim, Larysa Baraban, Gianaurelio Cuniberti

**Affiliations:** 1 Max Bergmann Center of Biomaterials and Institute for Materials Science, Technische Universität Dresden, 01069 Dresden, Germany; julian.schuett@nano.tu-dresden.de (J.S.); bcanton@nano.tu-dresden.de (B.I.); g.cuniberti@tu-dresden.de (G.C.); 2 Technische Universität Dresden, Center for Advancing Electronics Dresden, 01062 Dresden, Germany; 3 Department of Creative IT Engineering, Pohang University of Science and Technology, 37673 Pohang, Korea; hacle.arty@gmail.com; 4 Dresden Center for Computational Materials Science, 01062 Dresden, Germany

**Keywords:** silicon nanowire-based field-effect transistor, nanosensor, droplet-based microfluidics, point-of-care diagnostics, enzymatic reaction, β-galactosidase assay, lab-on-a-chip

## Abstract

Here, we present a miniaturized lab-on-a-chip detecting system for an all-electric and label-free analysis of the emulsion droplets incorporating the nanoscopic silicon nanowires-based field-effect transistors (FETs). We specifically focus on the analysis of β-galactosidase e.g., activity, which is an important enzyme of the glycolysis metabolic pathway. Furthermore, the efficiency of the synthesis and action of β-galactosidase can be one of the markers for several diseases, e.g., cancer, hyper/hypoglycemia, cell senescence, or other disruptions in cell functioning. We measure the reaction and reaction kinetics-associated shift of the source-to-drain current *I_sd_* in the system, which is caused by the change of the ionic strength of the microenvironment. With these results, we demonstrate that the ion-sensitive FETs are able to sense the interior of the aqueous reactors; thus, the conjunction of miniature nanosensors and droplet-based microfluidic systems conceptually opens a new route toward a sensitive, optics-less analysis of biochemical processes.

## 1. Introduction

The interest of the scientific community on the miniaturized lab-on-a-chip systems and on the label-free sensors has been dramatically growing during last two decades, due to the great promise of the technology to be efficiently used for a wide range of applications, e.g., molecular biology, proteomics, cell biology, (bio)chemistry, and even in everyday medical practice [1,2,3]. With the evolution of personalized medicine techniques, the role of early diagnostics experiences increasing development [4].

Commonly, the disease is directly associated with the immune response of the organism and accumulation of the respective biomarkers in blood, saliva, urine, etc. [4,5,6,7]. Along with disease development, the concentration of this specific biomolecules is also increasing [8,9]. A critical challenge is to reach the detection of extremely low concentrations of the biomarkers for life threatening cases, i.e., for cancer [10] or cardiac disease [11]-related antigens that enables detection at the very early stage. Additionally, apart from the ordinary sensing in the presence of a target substance, the monitoring of biochemical processes and reactions, as well as their kinetics, are of great significance [12]. Among multiple sensing techniques, label-free systems offer reduced costs of the assays along with the simplification of the measurement preparation, thus minimizing the influence on the real sample during screening [13,14]. Currently, all-electric sensing techniques give a promise to represent a simplified setup, compared to its optical counterpart that requires integration of light sources and detectors. To name a few, impedimetric [15], magnetic [16], potentiometric, e.g., field-effect transistor-based (FET) [17], amperometric [18], and micromechanical [19]. One of the challenges was to broaden the range of sensitivity of the sensors, since the concentration of the target substance is often directly dependent on the stage of the disease’s development. For this, a high-sensitivity recognition of sensors implemented with nanostructures was achieved, on one hand by signal and target amplification via an increase of the surface-to-volume ratio providing more binding sites and vacant places to react with the functional groups immobilized on the nanostructures of the sensor [20,21], and on the other hand by modulating the electrical behavior of the complete bulk of nanostructures compared to modulating only the surface of a planar material [22]. Implementing nanostructures, e.g., 1D and 2D (graphene sheets [23], nanorods [24], nanowires [25], beads [26], etc.) into a sensor design allowed for a greatly increased sensitivity of the (bio)chemical assays, in comparison to the macroscopic architecture. 

When integrated into the microfluidic format, it helps to gain the throughput of the analysis, while extremely reducing the volume of the sample needed [27]. Such a miniaturized packaging enables a high level of automation along with the ability to parallelize measurements (multiplexing, coupling with circuit boards, etc.) [28]. More specifically, it has been demonstrated that the nanomaterial-based FET sensors, integrated into microfluidic channels [25], represent an efficient asset to the diagnostic platform. Such sensors help to achieve chemical information (ionic, pH), reaction kinetics, or chemical process dynamics through associated changes of the surface potential at the transducer. Since the majority of the aforementioned techniques are aiming at biological and chemical sample analysis, movement toward the reduction of a sample’s volume and at the same time an increase in the reliability of the results are desired. Toward tackling the challenge of sample treatment and delivery to the sensor area, droplet microfluidics is an intrinsic part of the lab-on-a-chip systems, providing the ability to handle large amounts of micro-reactors with high flexibility in design, allowing the operation of droplets (generation, mixing, merging/breaking, sorting, encapsulation of research subjects, etc.) [29,30,31,32,33] down to pL-nL volume. The combination of droplet microfluidics with electrosensing techniques such as electrochemical impedance spectroscopy (EIS), field-effect measurements, waveguide-based techniques, amperometry, cyclic voltammetry, etc. would allow obtaining different information to be applied in a broad range of applications [34,35,36,37,38,39]. However, the implementation of nanostructures on the sensing areas exploiting their high sensitivity is expected to further enhance the power of these miniaturized sensors. Implementing them in a potentiometric system (e.g., FET) would be of extreme advantage for accessing a new information channel in the assay (e.g., ionic, pH). This was demonstrated for the first time using silicon nanowires (SiNWs) as the semiconductor material of the FETs, with the preliminary monitoring of pH changes caused by enzymatic glucose oxidation activity [27]. Interestingly, unlike glucose oxidase activity, some enzymes do not cause a change in pH, which supposes a challenge for measurements in FET format. On the contrary, β-galactosidase hydrolyses lactose into galactose and glucose, with no proton production [40], but possible changes in the total ionic content are detectable a priori. This enzyme has been related to Krabbe’s disease [41], GM1-gangliosidosis [42], or cell senescence [43], where activity units (U) in the range of 0.1–4 U are typically measured [44,45].

**Figure 1:**
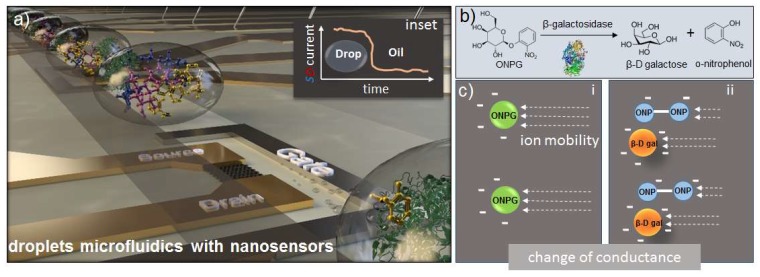
Concept of the work. (**a**) Schematic of the platform comprising a field-effect transistor chip as a sensor and a droplet microfluidics flow-cell as a sample delivery module. (**b**) The enzymatic reaction of β-galactosidase and ortho-nitrophenyl-β-galactoside (ONPG). (**c**) Ionic composition before and after the reaction, the arrows represent ion mobility. Field-effect transistor (FET) devices are located directly under the microfluidic channel allowing the detection of droplets one-by-one upon passing. Droplets act as a modulating liquid gate that leads to the tuning of the FET source and drain current.

Here, we present a system aiming at the monitoring of β-galactosidase activity in terms of ionic change of the media, including changes in the amount of charged species during a reaction where the pH remains stable. For this, we fabricated a microfluidic flow-cell that allows controllable droplet generation, mixing of the reactants directly on-chip, and guiding the droplet reactors directly to the sensor area (schematics in Figure 1). The flow cell was permanently attached to a FET chip and aligned with respect to the sensor’s location on-chip. Thus, we were able to monitor a single FET response with dependency on ionic changes upon enzymatic reaction with ortho-nitrophenyl-β-galactoside (ONPG) substrate concentrations (Figure 1b,c) in a label-free manner, tracking the kinetics of β-galactosidase/ONPG enzymatic reaction. In addition, the mobility of the ions within the solution before and after reaction differs (Figure 1c), due to the cleavage of ONPG into ONP and galactose.

## 2. Materials and Methods

### 2.1. Fabrication and Characterization of the FET Devices

**Figure 2:**
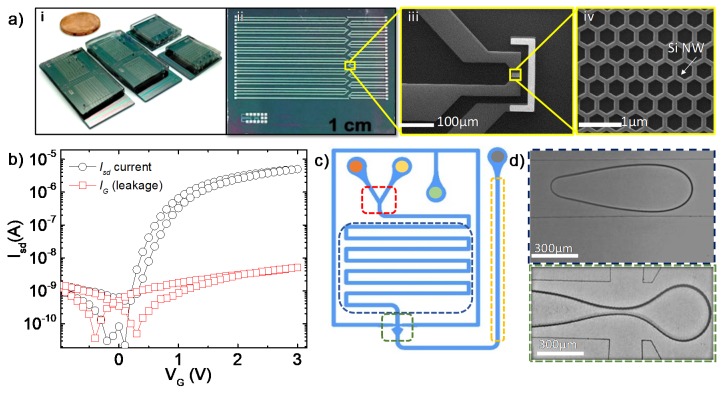
FET sensors with microfluidic integration. (**a**) Ready to use devices where a polydimethylsiloxane (PDMS) flow cell is permanently attached to the FET chip with the microfluidic channel aligned directly over the array of FETs: (**i**) FET chip with 16 devices (**ii**), SEM magnification of a single FET with Ag/AgCl-modified gate electrode (**iii**), and Si honeycomb structure (**iv**). (**b**) Transfer characteristics of a single FET device (@V_sd_ = 0.1 V). (**c**) Design of the microfluidic cell: two inlets for reactants (orange and yellow circles), inlet for oil (green circle), and outlet (gray circle). Reactants mix in the red area and the mixture is incubated while passing the channel (blue area). Droplets are generated (green area, magnified below) and guided to the sensors (yellow area). (**d**) Images of the channel (top) and droplet generating structure (down).

For the experiments, we developed a chip containing 16 SiNW field-effect transistors aligned for their convenient integration within the microfluidic channels, as demonstrated in Figure 2a. Each of the fabricated devices consists of source and drain silver electrodes interconnected by the SiNW channel and an Ag/AgCl-modified reference electrode (RE). The SiNWs were fabricated on a silicon-on-insulator (SOI) wafer consisting of a 100-nm thick As-doped top-silicon layer with a doping concentration of 10^17^ cm^−3^ using electron beam lithography and plasma etching. A honeycomb pattern with a width of 50 nm per nanowire and a semiconducting channel area of 12 × 30 μm was defined to take advantage of the mechanical stability and improved sensitivity of such a shape [46]. We used stacked Al_2_O_3_ (10 nm) and SiO_2_ (2 nm) layers as gate oxides, profiting from the high properties of the first one and the larger availability of hydroxyl groups on the second one, responding to ionic changes in the sample [47] (see the Supporting Information (SI) for additional details). Nanowire FET devices reveal *n*-type behavior, with negative charge carriers that respond with increasing current upon applying a growing positive gate voltage (see Figure 2b). A 300 μm wide and 15 μm high polydimethylsiloxane (PDMS) channel was produced using the soft lithography technique (see SI) and mounted on the chip (Figure 2a) by plasma bonding (Zepto, Diener Electronics, Ebhausen, Germany) with tight sealing to prevent undesirable solution leakage or evaporation, particle contamination, and liquid vibration due to air flow [48].

### 2.2. Microfluidic Chip

The design of the microfluidic cell (Figure 2c) fabricated by soft-lithography (Figure S1, Supporting Information, SI) consisted of two inlets for reactants (orange and yellow dots), an inlet for oil (green dot), and an outlet (grey dot). Once the reactants were mixed (red area), the solution was incubated while passing the channel (blue area). Droplets were generated (green area) and guided to the sensor (yellow area). Water-in-oil (mineral oil, Sigma-Aldrich, Munich, Germany) emulsion was used. In order to improve the surface interaction and stabilize formed droplets 2% of Span 80 (Sigma-Aldrich, Munich, Germany), surfactant was added to oil solution. The dependence between injection rates of oil and water phases versus the volume of generated droplets is presented in Figure S2 (SI). In the presented experiments, the droplet generation frequency was in the range of 1–2 Hz. The injection and guidance of the fluids were controlled with three syringe pumps (neMESYS, Cetoni GmbH, Korbußen, Germany). Fluorinated ethylene-propylene (FEP) capillary tubes (0.8 mm × 0.25 mm, Dolomite, Royston, UK) were plugged in all inlets connected with the syringe to deliver the reactants to the chip. The signal from the FETs was obtained using a Keithley 2604B source measuring unit (Tektronix GmbH, Cologne, Germany). 

## 3. Results

### 3.1. Measurements

**Figure 3:**
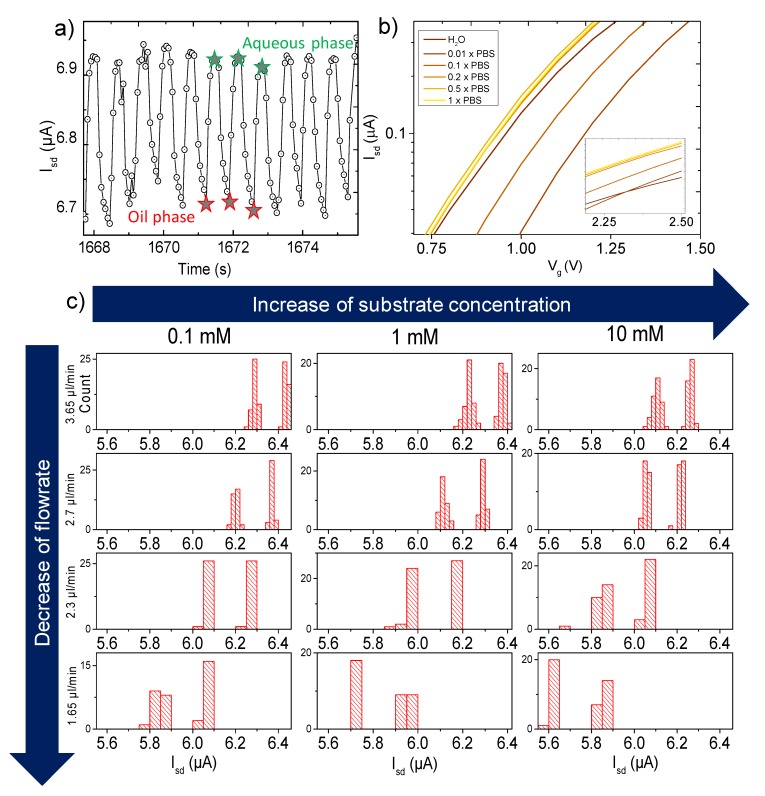
FET response to variations in sample composition. (**a**) *I_sd_* signal change with passing droplets. Local maxima correspond to every single droplet, while minima correspond to the oil separating them. (**b**) Transfer characteristics response to phosphate-buffered saline (PBS) buffer ionic strength variations. The calibration of the flow-rate test is shown in (**c**).

The exemplary time-domain measurements of the source-drain current I_sd_ modulation upon pumping of the droplets sequence is represented in Figure 3a. The local maxima of the current correspond to the aqueous droplet phase, containing the mixture of the reactants (dissolved in respective media) and the local minima of the current represent the signal of the oil phase. The FET was seen to respond to variations in ionic strength as well, with changes in the transfer characteristics in the presence of phosphate-buffered saline (PBS) at different dilutions in the range from 1× to 0.01× (Figure 3b). 

An additional standard test to verify and calibrate the function of the FET as the ionic sensor was done by measuring droplets with changing pH values. Several solutions of PBS in the range from pH 4 to 8 were prepared and investigated separately by fixing the gate voltage (V_g_) at 1.3 V and measuring the current (@ V_sd_ = 0.1 V). V_g_ = 1.3V was chosen as working voltage due to the highest slope of the transfer curve, thus at the level of the highest sensitivity. Before each run, the flow cell was completely flushed with mineral oil. The overall flow-rate value in this experiment was 1.65 μL/min (giving a frequency of droplet generation of approximately 1 Hz). The pH of the mineral oil (2% Span 80 as a surfactant) was 5. Merged data obtained from different pH solutions are presented in Figure S5a (SI) with evident dependence between the pH and source-drain current values. A sensitivity of 0.2 µA/pH was determined.

### 3.2. Enzymatic Reaction

β-galactosidase catalyzes the hydrolyzation of lactose into β-galactose and glucose. β-gal is an important enzyme of the glycolysis metabolic pathway, and it can be one of the markers for several diseases e.g., cancer development, hyper/hypoglycemia, cell senescence, or other disruptions in cell functioning. In order to conduct experiments to define the activity of in vitro β-galactosidase, lactose can be exchanged with the synthetic analogue ONPG for colorimetric detection. The mechanism of action of the enzyme on ONPG is the same as on lactose, but instead of glucose and galactose, the products are ortho-nitrophenol (ONP) and galactose. ONPG is normally colorless. When hydrolyzed by β-galactosidase, the resulting ONP shows a yellow color with the absorbance peak at 420 nm, which can be used as a colorimetric indicator of enzyme activity. Compared to similar molecules such as para-nitrophenol, ONP forms strong intramolecular H-bonds between hydroxyl and nitro groups, which prevents deprotonation and therefore the pH will not change [49]. However, the net ionic content will vary, considering the increased amount of hydroxyl groups (electronegative) before and after the reaction, giving rise to changes in the conductive properties of the sample. 

### 3.3. Spectrophotometry as Reference Test

The reaction was optically monitored as a reference to better understand the reaction kinetics. The light absorbance at 420 nm was measured during the gradual development of its characteristic yellow color (see Figure S3a), which follows the Michaelis–Menten kinetics [50]. The increase of β-galactosidase concentration increases the speed of the reaction, while the levels of ONPG affects the saturation point since it defines the amount of the substrate that can be processed by the enzyme. When the enzyme amount was kept constant at 1 U (Figure S3b), saturation was observed after 10 min for the smallest ONPG concentration (0.5 mM) (with the low absorbance level ca. 0.7 A.U.). No saturation was observed yet for higher ONPG concentrations, even after 10 min of reaction for 2 mM of ONPG at 2.0 A.U. levels (data not shown). On the contrary, when the substrate concentration was kept constant (1 mM) but the enzyme amount varied (Figure S3c), a very fast saturation was observed, e.g., for the highest tested enzyme concentration (20 U). Low enzyme concentrations resulted in a slower reaction kinetics, respectively. While potentially the three measurements with the same ONPG concentration should reach the same final absorbance, a longer time is needed for low enzyme concentration. 

In the following, we put forth to understand the contribution of the charged species produced during the reaction for the delivering of the “all-electric” curve of the β-galactosidase-driven enzymatic reaction. To do this, we use conventional measurements of the pH and the solution conductivity to determine the major “*influencer*” in the reaction. The results are summarized in Figure S4, SI. As expected, the pH measurements during the reaction time (from t = 0 s to t = 5 min) using pH paper and the conventional pH meter did not result in the measurable change of acidity from the nominal value of 7 (data not shown). In contrast to this, conductivity measurements with a standard conductivity meter (LF 330, WTW, Weilheim, Germany) showed the quick drop of the conductivity, which became more pronounced with an increase of concentration of both ONPG and β-galactosidase (Figure S4), evidencing changes in ionic strength. However, overall, the total amount of negatively charged ions increases during the enzymatic reaction, which should contribute to the carrier (electron) depletion of the n-type FETs using in this work, therefore reducing its conductivity.

### 3.4. FET Monitoring of β-Galactosidase Assay

β-galactosidase and ONPG were prepared at concentrations of 1 and 0.1 U/mL, and 10 and 1 mM, respectively. In order to understand the kinetics of the reaction, different time points of the reaction were measured. Once β-galactosidase and ONPG solutions were mixed, the solution was incubated, and droplets were formed. Thus, all the droplets that were forming at a certain flow rate reached the sensor at the same time, since the distance from droplet formation to the sensor remained the same. The frequency of droplet formation was about 1 Hz; thus, for each value of flow rate (and respective reaction time point), a signal from 300 to 600 droplets was collected. The results were analyzed by taking into consideration the effect of the flow rate on the signal. It is well known that one of the drawbacks of all platforms comprising microfluidics and ion-sensitive devices is the effect of flow stream, which perturbs the electrical double layer and thus the potential at e.g., the nanowire. In general, this phenomenon is causing the shift of the source-drain current (*I_sd_*) depending on the velocity of the liquid passing over the sensor. Since we are varying the flow rate in order to change the reaction time before the droplets (reactors) reach the sensors (see Figure 3, panel C), the effect of the streaming potential on the measured *I_sd_* need to be compensated. Panel C shows the statistical analysis of all local minima and maxima that correspond to the droplets at any fixed concentrations of the reactants. In order to eliminate the effect of the streaming potential, solutions can be found elsewhere in the literature [51]. In our case, the quantitative subtraction of the effect was the solution. For this, a streaming potential calibration was done, comparing the shift of the *I_sd_* at different flow rates and substrate concentrations in the absence of the enzyme. The results are depicted in Figure S5b, SI. Higher flow-rate values tended to higher *I_sd_*. The corresponding baselines were extracted and further subtracted from the assay experimental data, thus eliminating the effect of the streaming potential and making the data comparable at any flow-rate values. Such design of the experiment allowed obtaining a better understanding of the drift of the signal and provided a good timeframe for signal stabilization. Since lower values of flow rate increase the time gap between the droplet formation and detection, the amount of cleaved ONPG in each droplet was also increasing. 

## 4. Discussion

**Figure 4:**
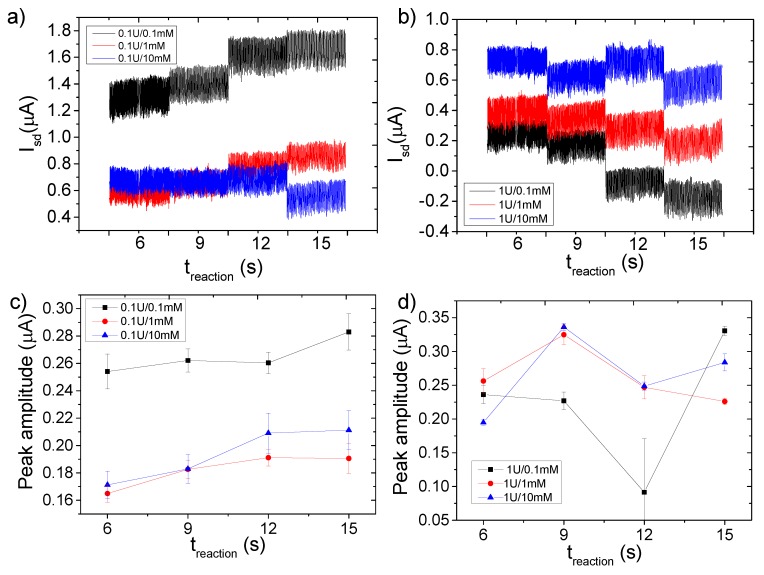
Data obtained from the assay with the subtracted effect of streaming potential. (**a**) Reaction with increasing substrate concentration and constant enzyme concentration at (**a**) 0.1 U and (**b**) 1 U. The reaction was detected at four time points. (**c**,**d**) show the averaged data from each respective set of experiments, calculating the difference between the maxima and minima of the peaks.

**Figure 5:**
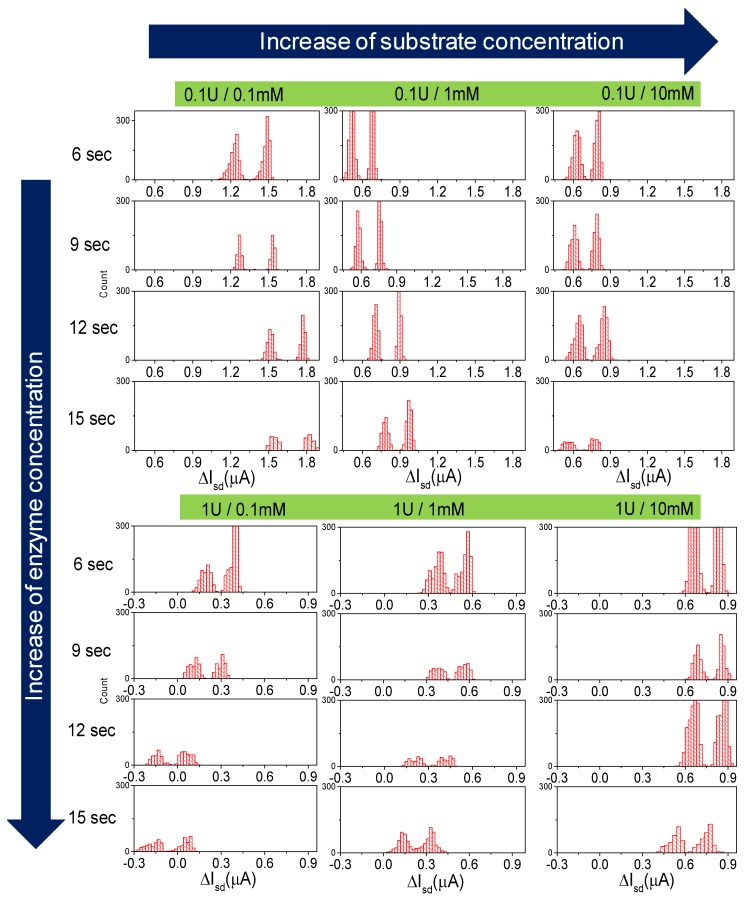
Kinetics map of the reaction for different time points, concentrations, and peak values.

According to the calibrations and theoretical calculations, the ionic charge of the media should cause the shift of the *I_sd_* during the reaction. After two sets of experiments with different concentrations of the enzyme and subtracting the effect of the streaming potential, the shift of the *I_sd_* was observed. In case of [β-galactosidase] = 0.1 U (Figure 4a), a current drop in dependency on [ONPG] was observed, with a strong difference between 0.1 and 1 mM ONPG (ca. 1.6 versus 0.70 µA drop), while the signal drop was saturated at higher concentrations (without a significant difference between 1 and 10 mM). However, the absolute current level was higher for the smallest [ONPG]. We also observed a tendency to change the slope direction at the highest concentration (10 mM) during the reaction. For the second case, [β-galactosidase] = 1 U (Figure 4b), we observed the opposite effect: the initial *I_sd_* level increased in dependency of [ONPG], along with the kinetics which keeps changing the slope in a negative direction proportional to [ONPG]. The difference between the maxima of the water and the minima of the oil is presented in Figure 4c,d. In the respective statistical analysis of the droplets’ content, which is represented as histograms (Figure 5), one can observe the shift of the source-drain current along with the change of the ratio with a decrease of flow rate. The sub-family of the curves in the top of the figure describes the shift of the signal upon changing the concentration of the substrate at a constant concentration of the enzyme (0.1 U). A lower set of panels represents the same shift of source-drain current, which is measured at a higher concentration of enzyme (1 U).

The two separate peaks in all panels of Figure 5 represent the baseline signal for the two phases: oil and water, respectively. Thus, the left peak represents *I_sd_* measured at the oil phase, and the right peak represents the signal from the sample in the water phase. The distance between peaks represents the height of the peak of droplets.

The distance between peaks represents the height of the peak of droplets. The counts represented in Figure 5 show that the amount of droplets appeared at the same Isd value and binned accordingly. Overall, the amount of droplets in bins for each flow rate and concentration varied from 300 to 1000. 

We suggest that this phenomenon is associated with the process of re-equilibration of the PBS buffer. The speed of buffer re-equilibration is directly dependent on the concentration of the enzyme and thus the amount of the processed substrate. Since local changes of ionic composition and charge carriers take place during the reaction, the buffer due to its purpose tends to compensate for the change. Thus, for lower enzyme concentration, the trend is explained by the higher velocity of buffer re-equilibration than the velocity of the enzymatic reaction. 

Peak analysis visualizes changes in several characteristics of the reaction in time. Firstly, the change in peak ratios, i.e., the ratio between the signal induced by media (reaction) phase and an oil phase, represents the change of the ionic composition of the media at a certain time point, allowing the comparison of different concentrations of the compounds. In addition, putting the aforementioned data together provides a visual interpretation of the general shift of the current level, which is mostly affected by the formation/decomposition of the new charged species due to the enzymatic reaction. During the reaction, β-galactosidase cleaves ONPG, degrading it into ONP (observable in yellow in spectrophotometry) and galactose. Thus, the final concentration of the charged groups is dependent on the initial concentration of the ONPG, but the kinetics (velocity) of the reaction is mostly affected by the concentration of the enzyme. The kinetics of the reaction depends not only on the enzyme and substrate concentration, but they also depend on the volume of the reacting solution as well. For larger volumes, the reaction tends to have a steep slope of the kinetics curve at the beginning of the reaction. Once the initial available amount of the substrate in small volume dV is cleaved by the initially available enzyme molecule, further reaction events will continue according to the diffusion of molecules within the solution. For smaller volumes, i.e., droplets V~nl, the molecule diffusion is confined in a tiny space, and mixing is enhanced by chaotic advection as the droplet travels through the serpentine channel [52], allowing a faster detection within the first few seconds.

## 5. Conclusions

We demonstrate a label-free method for monitoring the kinetics of the enzymatic reaction of β-galactosidase and ONPG in emulsion droplets with the possibility of high-throughput processing using an integrated honeycomb-based FET. We investigated the reaction with increasing ONPG concentration at various fixed enzyme units. The reaction, which was confirmed as well by optical determination, did not result in any pH differences. Since the pH remains constant during the reaction, a change in conductivity due to the decomposition of ONPG molecules was the addressed property. Furthermore, the effect of the streaming potential on the shift of source-drain current was analyzed in order to eliminate its influence on the signal caused by the ionic change of the reactive media. The designed microfluidics enabled mixing of the reaction compounds directly on-chip and provided an opportunity to obtain a signal from individual droplets during the first seconds of the reaction at low concentrations of the enzyme. Moreover, high statistical output for the analysis (approximately 600 droplets) for each particular concentration and flow rate provided high reliability of the obtained data. The droplets rate, as well as the signal acquisition rate, can be increased to improve the throughput of the analysis. We chemically probe the content of every single droplet in a row as independent events and resolve the ionic strength of the media, resulting in a change of a source-drain current *I_sd_* through the honeycomb structure. Finally, we demonstrated that the presented system is suitable for the label-free monitoring of enzymatic reaction in the absence of pH change. This opens the possibility of analyzing the “ion-based” profile of the biochemical process that can bring an additional information channel into the analysis.

## Supplementary Materials

The following are available online at https://www.mdpi.com/2072-666X/11/2/138/s1, Figure S1: Soft lithography for PDMS channel fabrication, Figure S2: Dependence between injection rates of oil and water phases (QH20/Qoil) versus the volume of generated droplets to estimate the volume of the reacting solution, Figure S3: Spectrophotometry test of the enzymatic reaction, Figure S4: Conductivity measurements of enzymatic reaction, Figure S5: The response of the FET to (a) droplets with different pH values and (b) variations in the flow rate in the presence of increasing ONPG concentration.
