# Supplementary material for: Nanosensors-Assisted Quantitative Analysis of Biochemical Processes in Droplets
Source: Micromachines (Basel). 2020 Jan 26;11(2):138. doi: 10.3390/mi11020138 (PMC7074628; doi:10.3390/mi11020138)
Supplement: Supplementary file 1 [file micromachines-11-00138-s001.pdf]

micromachines

Supplementary Materials: Nanosensors Assisted Quantitative Analysis of Biochemical Processes in Droplets

Dmitry Belyaev [1,*], Julian Schuett [1], Bergoi Ibarlucea [1,2], Taiuk Rim [3], Larysa Baraban [1,2,*] and Gianaurelio Cuniberti [1,2,4]

[1] Max Bergmann Center of Biomaterials and Institute for Materials Science, Technische Universität Dresden, 01069 Dresden, Germany; julian.schuett@nano.tu-dresden.de (J.S.); bcanton@nano.tu-dresden.de (B.I.); g.cuniberti@tu-dresden.de (G.C.)

[2] Technische Universität Dresden, Center for Advancing Electronics Dresden, 01062 Dresden, Germany

[3] Department of Creative IT Engineering, Pohang University of Science and Technology, 37673 Pohang, Korea; hacle.arty@gmail.com

[4] Dresden Center for Computational Materials Science, 01062 Dresden, Germany

* Correspondence: dmitry.belyaev@nano.tu-dresden.de (D.B.); l.baraban@hzdr.de (L.B.)

pH Analysis of Enzymatic Reaction

In order to eliminate the potential effect of pH cross-sensing, we monitored the pH during the reaction. 30 μL of β-galactosidase (final concentration 1 U/mL) was added to 5 mL of 10 mM solution of ONPG (in 0.1× PBS) and rapidly vortexed for better mixing. Further, at certain time points, 20 μL of the solution was dropped on pH paper, thus providing visual interpretation of the pH change during the reaction. The error relativity of the color change does not provide exact results since significant changes in the pH can be clearly detected. The same test was done using a standard potentiometric pH meter (Inolab Level 3 Terminal, WTW, Weilheim, Germany) to monitor the pH during 10 min reaction.

Conductance Analysis of Enzymatic Reaction

During the reaction, the amount of electronegative groups at the molecules is changing, leading to the change of the conductivity of the solution. Since no free protons are formed, one should not observe any change in pH, but the analysis of the reaction should be possible via monitoring of the conductance change.

Once ONPG is cleaved into ONP and galactose, the overall amount of ions is changing. The obtained data proved that the decrease in conductance was directly dependent on the concentrations of the enzyme and the substrate. For growing ONPG concentration at constant enzyme levels and vice versa, the value of conductance was showing a tendency to drop (Figure S4), with saturation starting to happen at only 1–3 min for high concentrations (0.1 mS/cm drop for 10–15 mM ONPG + 16.5–49.5 enzyme units, 0.3 mS/cm drop for 10 mM ONPG + 49.5 enzyme units). In order to understand the ionic changes, the reaction was investigated using a conductometer. The compounds were quickly mixed in Eppendorf tubes, the reaction started at this point and the tip of the device was immediately immersed into the solution. The experiment was conducted separately for constant concentrations of both reactants as well as the control measurements without the enzyme. The increase of ion clusters reduces their mobility97. Considering that ONP can form clusters100, and that multivalent and molecular anions taking part in this reaction have lower mobility compared to the monovalent atomic ions in the salts of PBS (mostly Na+ and Cl−), the increase of the fraction belonging to the first ones and their aggregate formation might be an explanation to the observed conductivity drop. The larger hydration radius of smaller ions (ONP versus ONPG) can also contribute to the lower mobility.

Flow-Cell Fabrication

Figure S1 shows the scheme of soft lithography process. PDMS was formed by mixing the base elastomer with the curing agent in a 7:1 (elastomer:curing agent) ratio, followed by degassing in a vacuum chamber. Once the PDMS was degassed, it was poured on an SU-8 resist mould fabricated by photolithography on a silicon wafer, using a steel frame to delimit the PDMS deposition area. After 1 h at 75 °C, the PDMS was completely polymerized. Then, the flow-cell was cut out with a scalpel and carefully removed from the master wafer. The inlets and outlets for tubings were made using a biopsy tool, providing very defined holes without damaging the structure of the fluidics design and flow cell by itself. As a result, the flow cell had negative pattern of the mask, thus providing channels when attached to other surfaces.

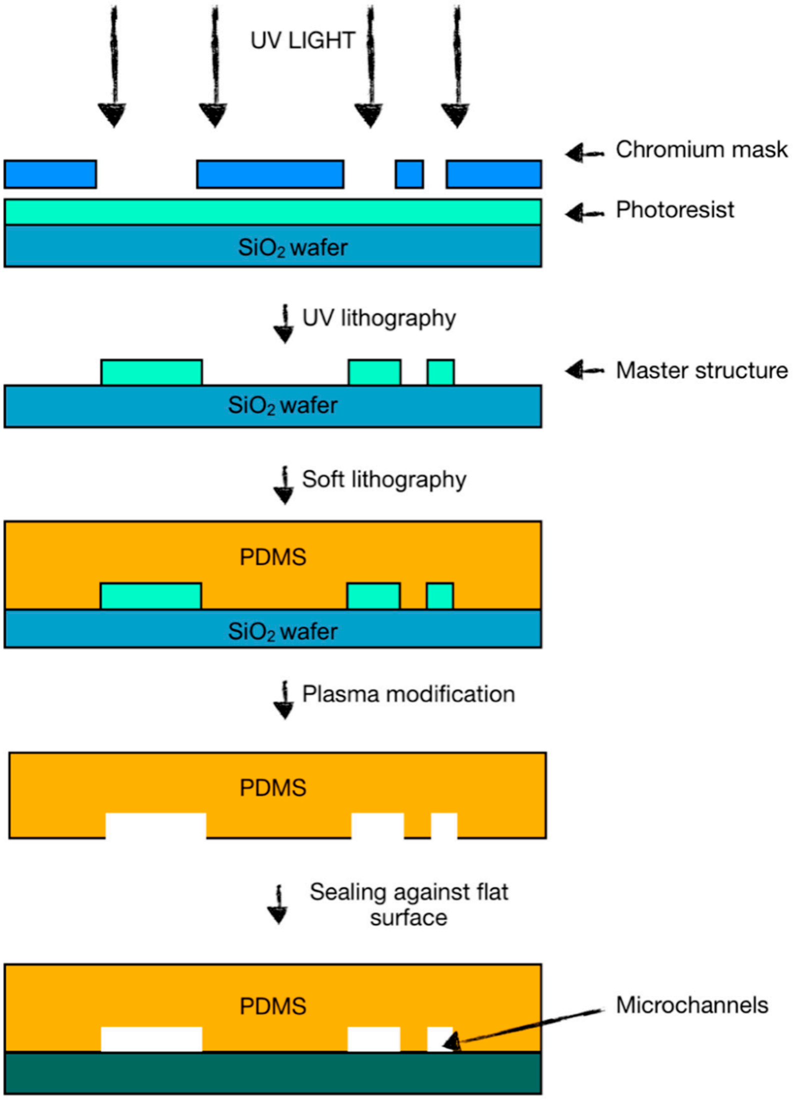

Figure S1. Soft lithography for PDMS channel fabrication. First, master structure is fabricated using photolithography at the silicon wafer, using SU-8 photoresist. Afterwards, the inverted PDMS mold was produced for fabrication of the microfluidic chip.

Chip Fabrication

The active region consisting on the source and drain electrode area and a honeycomb shaped structure based on nanowires of 50 nm width was defined using electron-beam lithography and ICP-RIE (inductively coupled plasma reactive ion etching) on an SOI wafer with 1017 cm^{-3} As doping. A standard photolithography process was carried out to pattern electrodes made by a stack of 200 nm

silver on 20 nm titanium, forming the embedded source, drain, and pseudo-reference electrode (pRE). Stacked Al_2O_3 (10 nm) and SiO_2 (2 nm) layers were deposited as gate oxides on the nanowire area. Finally, the electrode transmission lines were isolated with an additional 500 nm thick SiO_2 layer, leaving exposed the semiconductor area with the stacked dielectric layers, the pRE and the contact pads for electrical connection. For stable gate potential, the Ag was reacted with potassium chloride to form an AgCl layer covering the RE by electrochemical reaction applying 1 V for 1 min and using a Pt wire as a counter electrode. Fabrication process is described elsewhere in e.g., Kim et al. 2016.

Chip Characterization

Since the liquid phase is playing the role of a liquid gate, changes in ionic composition and concentration of charge carriers will affect the induced current. 1× PBS (10 mM phosphates, 150 mM ionic strength) was used as stock solution and then diluted with DI H2O to 0.5×, 0.2×, 0.1×, and 0.01×. The transfer characteristics of the FET were measured in presence of each PBS dilution in order to observe the response of the sensor to ionic strength.

Spectrophotometry

Solutions of β-galactosidase and ONPG were prepared at different concentrations. ONPG was diluted in PBS at pH 7 to concentration values of 0.5 mM, 1 mM, 2 mM, and 3 mM, with a final volume of 300 μL. Lyophilized powder of β-galactosidase from Escherichia coli (Sigma-Aldrich Chemie GmbH, Munich, Germany) was dissolved in deionized water (DI H2O) and diluted to concentrations of 3, 2, and 1 unit/mL with a final volume of 300 μL. 300 μL of the ONPG solution were pipetted in 1.5 mL disposable polystyrene cuvettes and placed in the holder of the spectrophotometer. Then 300 μL of β-galactosidase solution were added and the measurement procedure started immediately.

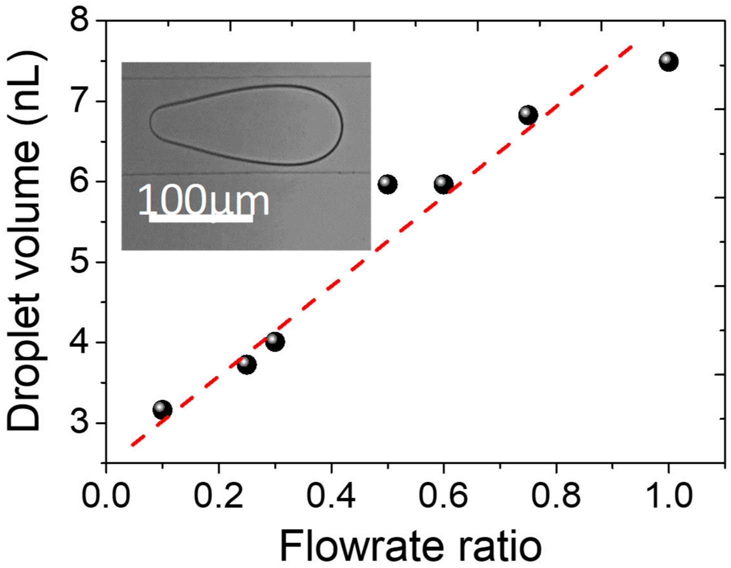

Figure S2. Dependence between injection rates of oil and water phases (QH20/Qoil) versus the volume of generated droplets to estimate the volume of the reacting solution. The inset shows the shape of a single droplet.

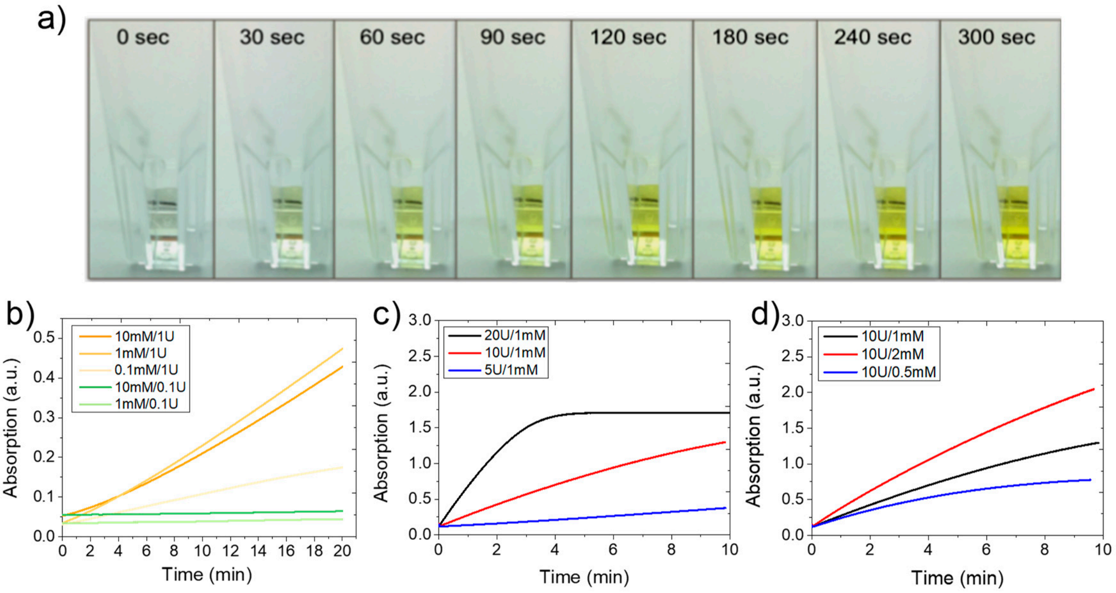

Figure S3. Spectrophotometry test of the enzymatic reaction. (**a**) Visual development of yellow color with time for [ONPG] = 1 mM and [β-galactosidase] = 10 U. (**b**–**d**) Kinetics test of the reaction at 420 nm wavelength for different concentrations of substrate and enzyme performed by spectrophotometer.

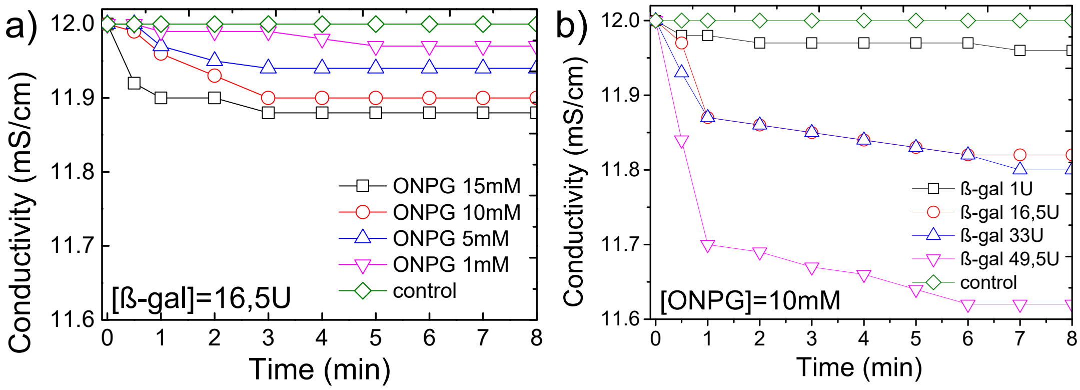

Figure S4. Conductivity measurements of enzymatic reaction with (**a**) constant enzyme and varying substrate concentration, and (**b**) vice-versa.

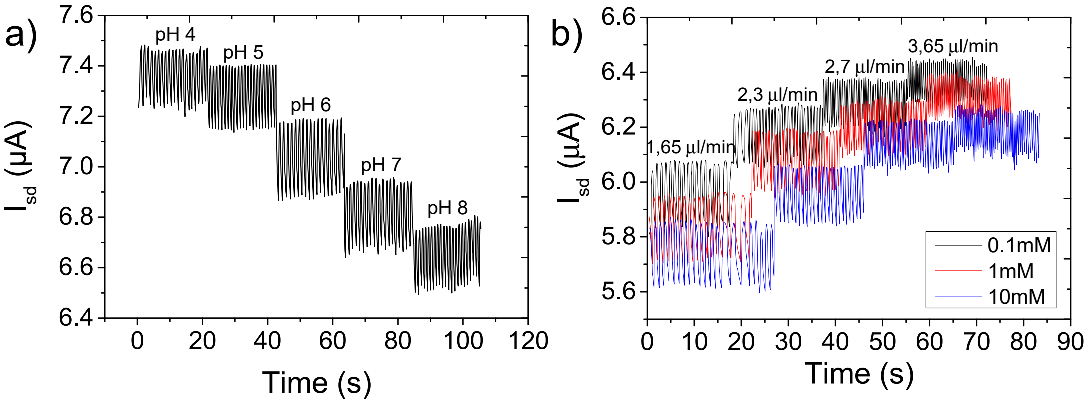

Figure S5. The response of the FET to (**a**) droplets with different pH values and (**b**) variations in the flow-rate in the presence of increasing ONPG concentration.
